# The Foreign Journals: Anatomy and Physiology

**Published:** 1839-07

**Authors:** 


### PART THIRD.

### Selections from the British and Foreign Journals.

---

### I. THE FOREIGN JOURNALS.

### ANATOMY AND PHYSIOLOGY.

*On the Disposition of the Blood-vessels in the Skin of Smallpox Patients.*
By Professor SEBASTIAN, of Groningen.

ERUPTIONS of the skin, properly so called, have their seat in the dermis, and are covered by a healthy transparent epidermis. Such are the various exanthemata and other pustular, vesicular, and papular eruptions.

In the short paper before us Dr. Sebastian's object is to investigate the cause why, in such eruptions, the epidermis is separated from the dermis only in certain circumscribed portions, to form pustules or vesicles, and to point out the abnormal changes which take place in the dermis. With this view he injected portions of the skin of patients who had died during the height of smallpox, with a penetrating injection, and submitted them to examination after being deprived of the epidermis, and otherwise prepared, generally by being dried, and saturated with turpentine.

He found the sites of the individual pocks highly injected, and the vessels forming a fine vascular network, corresponding in extent with the area of the pock, and separated from the skin on which no pocks had existed by two larger vessels, which encircled the site of the pock, and which, by giving off branches running towards a common centre, formed the vascular network. This vascular expansion may also be formed by branches derived from one vessel only, and the merging of several smaller into one larger expansion is the cause of confluent smallpox.

The result of this examination Dr. Sebastian considers as tending to remove the difficulties of the question. In consequence of the cause which produces smallpox a greater flow of blood takes place towards the skin, which by and by becomes inflamed, and the inflammation concentrates itself in certain circumscribed portions; these in a short time begin to secrete a serous fluid, which, as the inflammation increases, becomes purulent. The nature of the eruption depends, therefore, upon the vascular state of the dermis, but the cause which produces this peculiar vascular condition is as obscure as ever. Professor Sebastian leaves the question undecided whether the vascular network is a new formation, or whether it is the result of the greater injection of vessels already existing.

*Nat. Tijds.* i., 2. 1838.

---

*Observations regarding the Reproduction of the Lens.*
By Dr. PAULI, of Landau.

DR. PAULI, being of opinion that the experiments made as to the regeneration of the lens had yielded contradictory results, because animals of too small a size had been chosen for the operation, and extraction, the only mode of operating here admissible, had not been performed with sufficient care and circumspection, resolved to try what could be done by taking the larger domestic animals for the subject of his experiments. He accordingly extracted the lenses in an old hound

(Jagdhund) and in a bull. The former was killed 163 days, and the latter 211 days after the operations. "On examination I found," says Dr. Pauli, "in the right eye of the hound no trace either of capsule or lens. Behind the lower margin of the uvea there appeared to be some crystalline flakes; but I will not assert this positively. In the left eye there was in the situation of the lens a body similar to that which had been extracted, but softer and smaller; it was transparent. At the incision in the capsule the new lens was more flattened; the edges of the incision in the capsule appeared somewhat drawn into the new substance, and more firmly connected with it than in the rest of the circumference of the capsule. In both eyes of the bull I found nearly the same changes. The size of the new lens was not, however, much more than the half of that which had been extracted, but it was firmer and denser than that in the left eye of the hound—a circumstance which might be owing to the hound having been killed about two months sooner than the bull. The edges of the incision in the interior wall of the capsule were, as in the hound, apparently inclined towards the new lens, and less easily separable from it than at other places. Both animals retained vision until their death."

*Monatschrift für Medicin, &c. Jan. and Feb.* 1839.

---

*On the Episternal Bones.* By G. BRESCHET, Professor of Anatomy at the School of Medicine, Paris.

M. BRESCHET has discovered two new bones, which form part of the sternum, and are situated upon its superior and lateral part. In the young subject they are attached to the sternum by a synovial membrane and a fibrous capsule. They have somewhat the appearance of the pisiform bones, but of a greater size. In the adult these two episternal bones are anchylosed with the sternum, but the line of separation is distinct. These bones are not regularly rounded, but are flattened where they look towards the sternum.

Breschet considers these bones to be analogous to the episternal bone of the ornithorrynchus, ant-eater, and armadillo. The latter bone was described by Meckel, Rudolphi, and others: it articulates with the scapula. The episternal bone of the human subject is considered by M. Breschet to be the rudiments of the cartilage of the thirteenth rib, which is not unfrequently met with. It does not properly belong to the sternum, and is formed independently of it, but is soon anchylosed with it, as is the cartilage of the first rib. This episternal bone in the *Bradypus Tridactylus* is continuous with the sternum anteriorly, and posteriorly with the costal element of the seventh cervical vertebra; in this manner an additional rib is formed, and that at the expense of the last cervical vertebra, which has been generally considered as appertaining to the dorsal region.

*Annales des Sciences Naturelles. August,* 1838.

---

*Case of Spontaneous Rupture of the Spleen.* By Dr. NÜCKEL, of Cöln.

MR. J., æt. 25, had suffered for a fortnight from diarrhœa, but did not seem very ill, being able to get about. He took lead and opium, in small doses, without benefit. For two days before his death he kept his bed, on account of pains in the abdomen; on the last, he was suddenly seized with a feeling of anguish (Angstgefühl), and with cold sweats, &c.; he expired after a few hours. On examining the body, forty-eight hours after death, there was found a great effusion of blood in the abdomen and pelvis, which was traced to an angular rent in the spleen, three or four lines broad, and situated on the lower part of the anterior and outer surface of the viscus. The spleen measured about five inches in length and four in breadth. Its surface was of a dark, livid colour. Its coat was so rotten that the fingers pierced it in handling it; the parenchymatous substance resembled a dark red paste. The great vessels in the abdomen were sound. The stomach and upper portion of the duodenum were normal, but the ileum was covered with numerous ulcers. *Medicinische Zeitung. May* 8, 1839.

